# The “ART” of Epigenetics in Melanoma: From histone “Alterations, to Resistance and Therapies”

**DOI:** 10.7150/thno.36218

**Published:** 2020-01-01

**Authors:** Thomas Strub, Robert Ballotti, Corine Bertolotto

**Affiliations:** 1 Université Nice Côte d'Azur, Inserm, C3M, France; 2 Biology and pathologies of melanocytes, Equipe labellisée ARC 2019, C3M, team 1, France

**Keywords:** melanoma, epigenetics, drug resistance, targeted therapy, immunotherapy

## Abstract

Malignant melanoma is the most deadly form of skin cancer. It originates from melanocytic cells and can also arise at other body sites. Early diagnosis and appropriate medical care offer excellent prognosis with up to 5-year survival rate in more than 95% of all patients. However, long-term survival rate for metastatic melanoma patients remains at only 5%. Indeed, malignant melanoma is known for its notorious resistance to most current therapies and is characterized by both genetic and epigenetic alterations. In cutaneous melanoma (CM), genetic alterations have been implicated in drug resistance, yet the main cause of this resistance seems to be non-genetic in nature with a change in transcription programs within cell subpopulations. This change can adapt and escape targeted therapy and immunotherapy cytotoxic effects favoring relapse.

Because they are reversible in nature, epigenetic changes are a growing focus in cancer research aiming to prevent or revert the drug resistance with current therapies. As such, the field of epigenetic therapeutics is among the most active area of preclinical and clinical research with effects of many classes of epigenetic drugs being investigated. Here, we review the multiplicity of epigenetic alterations, mainly histone alterations and chromatin remodeling in both cutaneous and uveal melanomas, opening opportunities for further research in the field and providing clues to specifically control these modifications. We also discuss how epigenetic dysregulations may be exploited to achieve clinical benefits for the patients, the limitations of these therapies, and recent data exploring this potential through combinatorial epigenetic and traditional therapeutic approaches.

## Introduction

Incidence of cutaneous malignant melanoma is rising steadily. Its therapeutic management is a real challenge for oncologists as it is amongst the solid malignancies most refractory to conventional cancer therapies 1. Recently, our improved understanding of the molecular mechanisms underlying cutaneous melanoma (CM) biology has led to improved treatments for advanced CM, which includes targeting the MAPK signaling pathway which dramatically improved patient outcome. More recently, the use of immune checkpoint inhibitors have shown to be effective in almost a third of all patients 2. Moreover, improved overall survival outcomes were observed with targeted therapies in patients with *BRAF*^V600^ mutant unresectable stage III or stage IV melanoma, with up to 70% of patients who responded according to Response Evaluation Criteria in Solid Tumors (RECIST) along with tumor size reduction in 95% of patients in phase 3 randomized clinical trials 3-6. Unfortunately, these results are either transient or limited to restricted subsets of patients due to intrinsic or acquired resistance. What is certain is that both intrinsic and acquired resistances can be driven by genetic and epigenetic alterations underlying gene expression changes in genetically identical cells. Epigenetic reprogramming rewires metabolic and signaling networks, thereby driving the emergence of tumor cell subpopulations with distinct behavior and altered antigenic landscape 7. This intratumor heterogeneity drives new resistance mechanisms to escape drug cytotoxicity or surveying by the immune system, enabling tumor regrowth and disease relapse. As the side effects are often severe and can be life-threatening, alternative therapies must be explored. To advance in this field, we must understand the mechanisms of resistance in order to identify novel targets and therapeutic approaches for more effective and long-lasting treatments for patients.

In this review, we introduce the major advances in CM treatments and summarize recent discoveries of epigenetic influences. We focus on histone modifications, chromatin remodeling and histone variants in metastatic CM, followed by their role in resistance to therapy, and discuss why they are important therapeutic targets. We also discuss epigenetic changes, which are now receiving attention in metastatic uveal melanoma (UM), for which therapeutic intervention remains extremely limited. Additional important epigenetic events such as DNA methylation and non-coding RNAs are beyond the aim of this review. These processes will be briefly discussed and we refer the reader to other reviews 8,9.

## Epigenetic regulation

Recent advances in deciphering the mechanisms of melanoma progression underlined a critical role for epigenetic alterations, thereby turning both academic and medical attention towards the application of epigenetics to melanoma detection and therapeutics.

### Chromatin structure and histone modifications

By remodeling the chromatin structure, epigenetics co-operate with transcription factors and the translational machinery in fine-tuning gene expression 10. Chromatin is the physiological state of the eukaryotic genome, in which DNA is packaged with its intimately associated proteins, the majority of which are histones. The nucleosome is the basic repeating unit of chromatin, which consists of 146 base pairs of DNA wrapped around an octamer of histone proteins: two of each histones H2A, H2B, H3 and H4 and/or their variants 11,12. While chromatin is a highly organized structure, changes in its structure are essential for regulation of key cellular processes and therefore must be dynamic. Changes include post-translational modifications (affecting histones N-terminal tails, such as acetylation (ac), methylation (me), ubiquitylation, phosphorylation, sumoylation or glycosylation), ATP-dependent chromatin remodeling, and the incorporation of specialized histone variants into chromatin 11-14. As a biological consequence, the genome can be partitioned into distinct domains, such as euchromatin (where DNA is “open” allowing transcription) and heterochromatin (where DNA is “closed” preventing transcription). This dynamic process is driven by the activity of specific cellular enzymes, for example, histone methyltransferases (HMTs), histone demethylases and histone acetyl transferases (HATs)/histone deacetylases (HDACs) for determining the status of histone methylation and acetylation, respectively. The balance of these histone modifications orchestrates the above mentioned states by modifying the charges of the nucleosomal structure by respectively decreasing or increasing the histone-DNA interactions and therefore modulation of transcriptional activation and repression 15.

Histone methylation comes in different forms. Indeed, histone lysine (K) can be mono-, di- or tri-methylated and defines different regulatory regions according to their methylation status. For example, H3K4me1 was the first histone modification connected with distal regulatory regions, called enhancers, whereas H3K4me3 is enriched at active promoters 16. Of note, these regulatory elements are known to play a key role in regulating expression of genes important for maintaining cell identity and disease 17. In addition to its status, the methylation site on the histone tail is also critical for diverse functions; H3K79me2 or H3K36me3 are mainly found where active transcription takes place whereas H3K9me3 or H3K27me3 are linked to transcriptional repression 13. On the other hand, the histone acetylation state is also considered as a recruitment platform for transcription factors such as for bromodomain-containing proteins 15.

Another player in chromatin remodeling processes is the SWI/SNF complex (also known as BAF complex). This large multi-subunit complex uses the energy of ATP hydrolysis to remodel and evict nucleosomes at gene promoter, impacting the recruitment of regulators and therefore transcription regulation. This complex contains more than 15 members including an ATPase (BRG1, or BRM also known as SMARCA4 or SMARCA2 respectively) and a DNA binding domain subunit (ARID1A, ARID1B or ARID2) 18. Finally, with their sequences and structural variations from the canonical histones, histone variants can replace their counterparts within the nucleosome 12. Histone variants can have temporal and tissue-specific expression and affect a variety of DNA-templated processes.

Thus, by disrupting chromatin contact or by affecting the recruitment of nonhistone proteins to chromatin, all the above-mentioned reversible modifications orchestrated by “writers, readers and erasers” (enzymes that add, bind or remove chemical modifications to histones) influence many fundamental biological processes 19. Strikingly, it has become evident in the last decade that the epigenetic landscape contains a unique ability to regulate key cellular and developmental processes 13,20,21, and that its deregulation may contribute to melanoma initiation, progression and drug resistance that will be discussed hereafter.

## Epigenetic alterations in melanoma development and pathogenesis

Despite the unquestionable importance of oncogene activation and/or tumor suppressor inactivation in melanoma tumor burden, a growing body of evidence suggests that modifications in the epigenetic landscape drives the alteration of transcriptional programs that are tightly associated with the development of melanoma pathogenesis.

Regulation of chromatin in its various active states is largely controlled through post-translational modifications (PTMs) of the core histone proteins mediated by histone writers, erasers, and readers. Epigenetic regulations in melanoma, especially through these histone modifications, are gaining more and more attention. To begin with, insight into the importance of histone modifications in melanoma development emerged due to the fact that nevi, which are benign melanocytic lesions, mostly carry the oncogenic BRAF^V600E^ mutated form but rarely become malignant melanoma. This indicates that additional events are necessary to initiate melanoma. Patton *et al.,* developed the first animal model of a BRAF^V600E^ driven melanoma using a transgenic zebrafish model expressing the human BRAF^V600E^ under the control of the *mitfa* promoter. They showed that in a p53 deficient background, only a fraction of zebrafish develop melanoma tumors 22. As only a subpopulation of genetically identical cells promote melanoma, this fact highlights the importance of additional molecular events beyond genetic alterations. To assess this, the same group developed a p53/BRAF/crestin: EGFP zebrafish model. The crestin gene first marks the neural crest progenitors during embryonic development but importantly, it is re-expressed specifically in melanoma tumors in adult zebrafish allowing them to track melanoma lesions at the time of their initiation 23. Relevant in the scope of this review, they found H3K27ac super-enhancer marks *(*enhancer cluster regions) at the* sox10* locus, which plays a key role in neural crest formation and melanomagenesis, suggesting an epigenetic mechanism to increase SOX10 expression leading to the reemergence of the neural crest progenitor state to initiate melanoma 23.

### Histone modifications “Writers”

**Figure 1:**
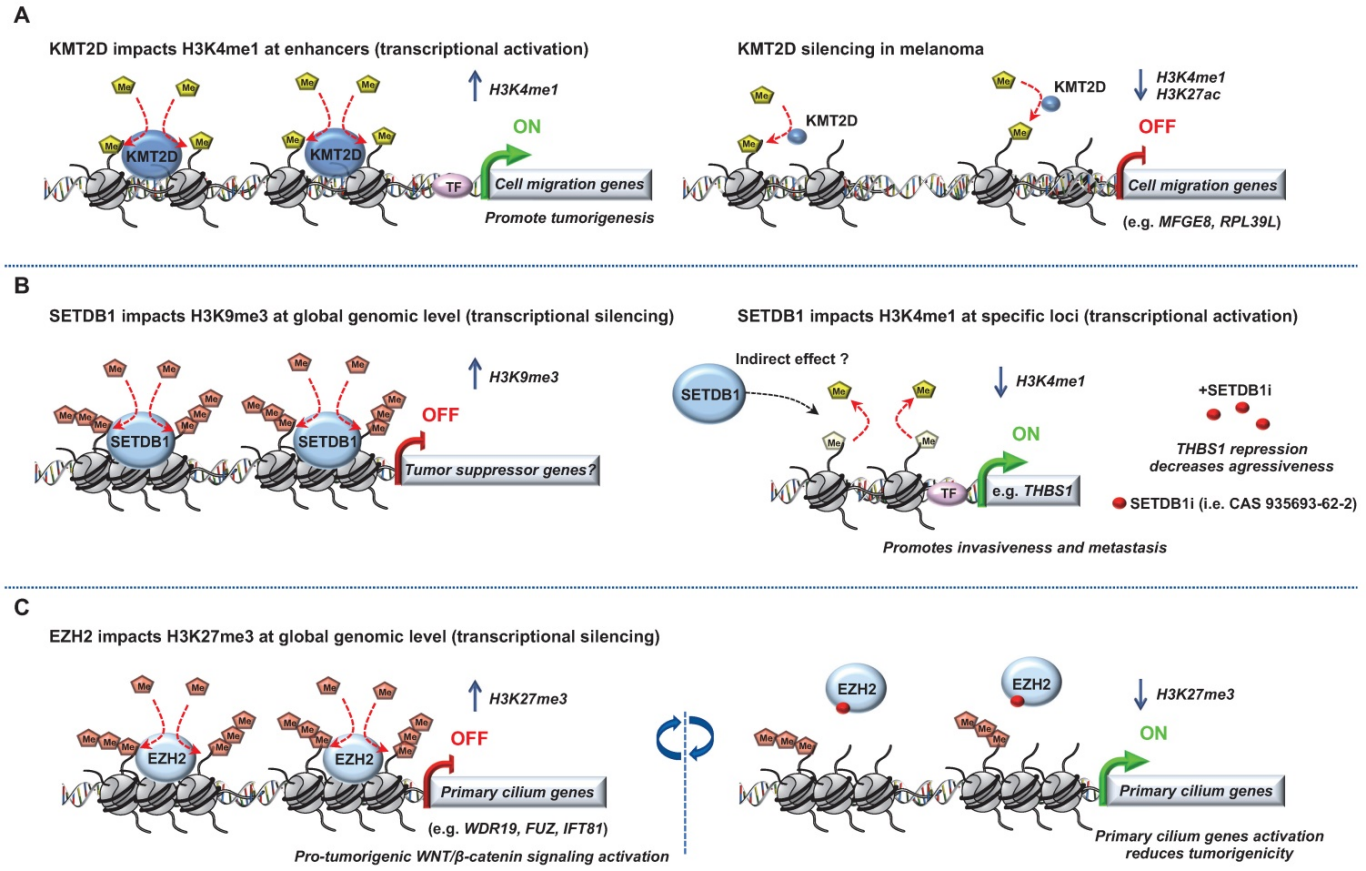
** Role of “Writers” in melanoma.** Epigenetic mechanisms driven by the histone lysine methyltransferases ((A) KMT2D, (B) SETDB1 and (C) EZH2) in melanoma progression. Few epigenetic players can be targeted by small molecules to reverse the chromatin state and decrease tumorigenicity.

Several studies have highlighted a role for “chromatin writers” in melanoma progression (**Figure 1**). Using metastatic melanomas from patient-derived tumors, Bossi *et al.,* performed the first *in vivo* genetic screen targeting chromatin players with specific shRNA libraries 24. Their study identified an unprecedented number of genes essential for tumor growth (e.g *BAZ1B*, *SMARCA4*, *CHD4*, *KMT2D*) and a certain interpatient heterogeneity. Importantly, these genes were not mutated in the same patients suggesting that the signaling pathways in these tumors are activated in a patient-specific manner. The authors focused on KMT2D, the major methyltransferase for H3K4me1 enhancer in mammals, implicated therefore in gene expression program 25. KMT2D-silencing leads to the inactivation of a subset of KMT2D-bound enhancers with a decrease of H3K4me1 and H3K27ac along with a down-regulation of genes which are critical for cell migration (e.g. *MFGE8* and *RPL39L*). Of note, the most proximal genes to these enhancers were KMT2D target genes suggesting that KMT2D deregulates enhancer activity to promote tumorigenesis 24. However, an important heterogeneity was observed within the patients analyzed, suggesting distinct signaling pathways involved, most likely reflecting tumor-specific environment or genetic context. Usually, best candidates for development of targeted therapies are genes harboring biologically relevant mutations. However, taking into consideration the patient heterogeneity and that most genes identified in that study critical for tumor maintenance are not somatically mutated, the clinical impact of this study is demonstrated by their increased number of potential druggable genes for each patient 24.

Another “chromatin writers” implicated in melanoma is the SET domain bifurcated 1 (SETDB1), a member of the SUV39 family of histone lysine methyltransferases, catalyzing methylation of lysine 9 on the histone 3 which leads to epigenetically mediated gene expression silencing 26. Interestingly, the deposition of H3K9me3 on histones by SETDB1 occurs upon its recruitment to methylated CpG islands *via* a methyl-CpG-binding domain 27. Linking DNA methylation with heterochromatin formation at specific loci suggest a precise transcriptional repression control for a more accurate gene expression program. Strikingly,* SETDB1* is amplified in human melanoma compared to nevus or normal skin and accelerates melanoma development in the same zebrafish BRAF^V600E^ model system described above 28. Recently, the study from Orouji *et al.,* unraveled a SETDB1-mediated epigenetic mechanism in melanoma progression. They showed that the activation of thombospondin-1 (THBS1), known to promote invasiveness and metastasis formation in melanoma, is induced by SETDB1. In this case, in addition to H3K9me3, SETDB1 alters the methylation patterns related to H3K4. Indeed, they identified enrichment for H3K4me1 upstream of the *THBS1* gene which was reversely influenced by SETDB1 expression suggesting that SETDB1 may act not only on regulating H3K9me3 distribution but also on additional epigenetic marks to impact gene activation or repression. Finally, treatment with a small molecule inhibitor for H3K9me-specific histone methyltransferase to block the SETDB1 protein significantly decreased melanoma cell viability. Of note, to temper the impact of other H3K9 histone methyltransferases, the authors focused on melanoma cell lines with high levels of endogenous SETDB1 only. Interestingly, melanoma cells with low levels of SETDB1 were not affected suggesting SETDB1 as a promising new therapeutic target in melanoma 29.

Another histone methyltransferase involved in melanoma is enhancer of zeste homolog 2 (EZH2), the catalytic subunit of the polycomb repressive complex 2 (PRC2) catalyzing trimethylation of lysine 27 on histone 3 subsequently repressing transcription. EZH2 expression is elevated and associated with poor survival in melanoma. Its conditional ablation inhibits tumor growth and metastases in a NRAS^Q61K^ melanoma mouse model 30. Conversely, the most common human EZH2^Y646N^ gain of function somatic mutation (Y641F in mouse) through H3K27me3 accumulation and gene repression, favors melanoma progression 31-33. EZH2 has been shown to exert its effect through stimulation of the noncanonical NF-kB pathway leading to senescence bypass 34 and epigenetic silencing of primary cilium genes that results in activation of the pro-tumorigenic WNT/β-catenin signaling 31.

A specific cooperation between Ezh2^Y641F^ and B-Raf^V600E^ but not N-Ras^Q61R^ in inducing melanoma in mice was also reported 33. Of note, the role of EZH2 and its associated change in histone trimethylation seems more complex than expected. Indeed, Souroullas *et al.,* showed that although Ezh2^Y641F^ triggers H3K27me3 accumulation, it also caused a vast reorganization of chromatin structure, including a loss of H3K27me3 that was associated with increased transcription at many loci 33. Together, the abovementioned studies have demonstrated that EZH2 function can be effectively inhibited by a number of small molecules reducing melanoma cell growth and metastases. The translation of EZH2 inhibitors into clinical trials have shown preliminary evidence of clinical response (CR) in cancer 35. Notably, a recent study identified a molecular mechanism linking MAPK signaling activation mediated by BRAF^V600E^ mutation and downstream H3K27me3 remodeling for maintenance of gene expression silencing in tumorigenesis. The authors demonstrated that c-Myc regulates transcription of the PRC2 complex components (i.e. Ezh2, Suz12 and Jarid2) as well as their post-transcriptional levels. This regulation mediated by c-Myc is essential for BRAF^V600E^-induced H3K27me3 deposition and gene silencing in tumorigenesis 36.

### Histone modifications “Readers”

**Figure 2:**
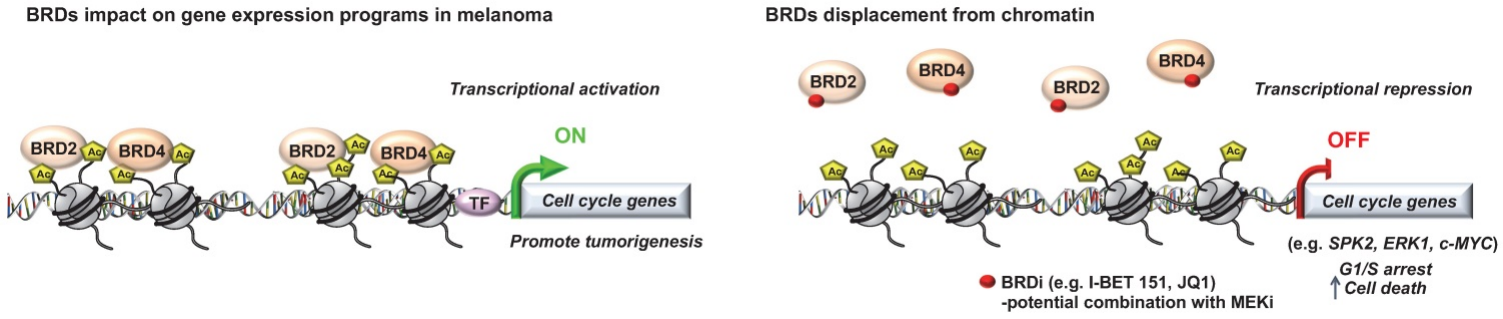
** Role of “Readers” in melanoma.** Key roles of the bromodomain and extraterminal domain (BET) proteins in melanoma progression that can be targeted to decrease tumorigenicity.

Critical role for a number of “chromatin readers” in melanoma has now been confirmed by several groups (**Figure 2**). These “reader” proteins are able to recognize a specific chromatin modification, subsequently initiating downstream regulatory processes. We will focus here on the bromodomain and extra-terminal domain (BET) proteins (BRD2, BRD3, BRD4 and BRDT), which bind to acetylated lysine residues of histone. Briefly, it has been shown that these proteins render nucleosomes marked by acetylation permissive to the passage of elongating RNA polymerase II, and therefore couple histone acetylation to gene expression regulation 37. Among this family, it has been shown that BRD2 and BRD4 are overexpressed in melanoma tissues and are required for tumor maintenance by controlling the expression of key cell cycle and survival genes. In particular, using the bromodomain (BrD) containing proteins inhibitor I-BET151, Gallagher *et al*., observed a selective inhibition of the NF-κB signaling pathway with genes involved in induction of inflammation (e.g. *VEGF*, *CCL-20*), cell cycle regulation (e. g. *CDK6*) and a downregulation of cytokines production such as IL-6 and IL-8 mainly *via* BRD2 displacement 38. The same group has also shown that I-BET151 inhibits melanoma growth *in vivo* and induces apoptosis which is caspase-dependent and associated with G1 cell cycle arrest in melanoma cells 39. Interestingly, by using the BrDi MS436 or MS417, another BrDi previously reported to have higher binding affinity and specificity for BET family members, similar observations (cytostatic effect along with G1 arrest) were reported 40. The authors showed that BET displacement downregulates the key cell cycle genes *SKP2*, *ERK1* and *c-MYC* along with the accumulation of cyclin-dependent kinase inhibitors (e.g. p21 and p27) 40. These studies suggest that specific inhibition of BET family members similarly impairs melanoma cells growth *in vitro* and *in vivo* than general BrDi. Moreover, transcriptomic analysis of melanocytes and melanoma cells exposed to the BET inhibitor JQ1 identified the transmembrane protein, AMIGO2, as a BET target gene essential for melanoma cell survival 41. The authors showed that *AMIGO2* is regulated by a melanoma-specific BRD2/4-bound promoter and super-enhancer configuration 41. Importantly, these studies reported that BETi efficacy was not influenced by BRAF or NRAS mutational status, supporting the inhibition of BrD proteins, especially BET family members, to modify epigenetic mechanisms of gene expression to correct disease states in patients for whom no effective targeted therapy is offered. On that note, an effective combination treatment for NRAS-mutant melanoma remains a therapeutic challenge in this field. A recent study used this model to test compound combinations to avert resistance. In that study, using a combination of BET and MEK inhibitors, they identified a critical role for the transcription factor TCF19 in cell cycle checkpoints regulation. TCF19 downregulation triggers a substantial transcriptional perturbation and activates pro-apoptotic signaling leading to cell death 42. Underlying the clinical aspect of these studies, the co-targeting of BET and MEK has been proposed in NRAS mutant melanoma cell lines or for melanomas with no other therapeutic options to offset resistance to targeted and/or immunotherapies 42.

### Histone modifications “Erasers”

**Figure 3:**
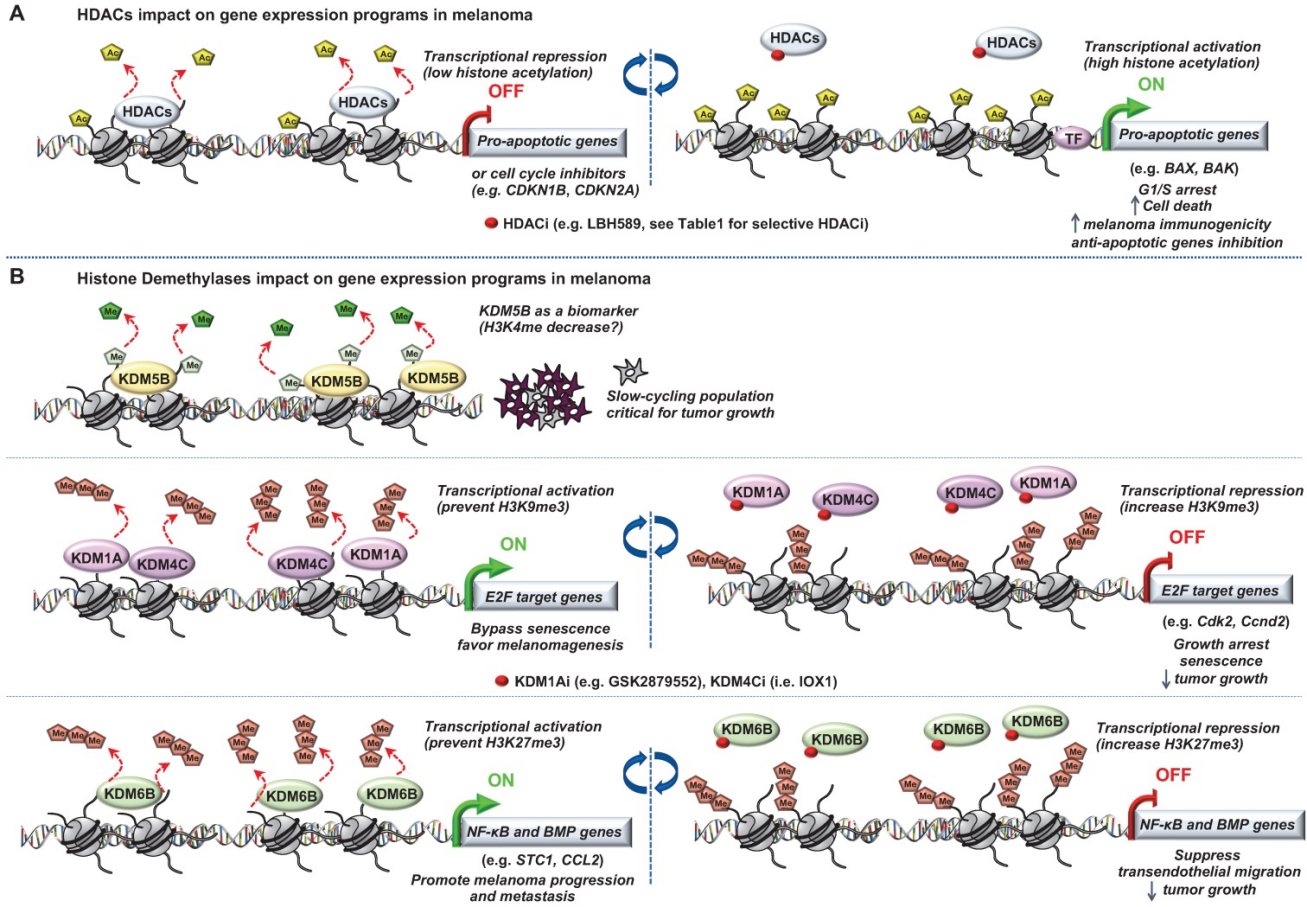
** Role of “Erasers” in melanoma.** Epigenetic mechanisms driven by (A) histone lysine deacetylases (HDACs) or (B) histone lysine demethylases (KDM5B, KDM1A, KDM4C and KDM6B) in melanoma progression. Few epigenetic players can be targeted by small molecules to reverse the chromatin state and decrease tumorigenicity.

The most studied of these modifying enzymes are the HDACs removing acetyl groups on the histone tails (**Figure 3**). Typically, HDACs belong to either the histone deacetylase family or the Sir2 regulator family. In humans, HDACs are divided into four classes. The class I proteins (HDAC1, HDAC2, HDAC3, and HDAC8) mostly nuclear, the class II proteins (HDAC4, HDAC5, HDAC6, HDAC7, HDAC9, and HDAC10) that shuttled between the nucleus and cytoplasm, the class III proteins (SIRT1, SIRT2, SIRT3, SIRT4, SIRT5, SIRT6, and SIRT7) that are the NAD dependent sirtuins and the class IV protein (HDAC11) 43. Studies of epigenomic alterations using non-tumorigenic melanocytes from nevi and tumorigenic melanocytes from melanomas revealed a loss of histone acetylation and H3K4me2/3 on regulatory regions proximal to specific cancer-regulatory genes involved in important signaling pathway-driving melanoma 44. Treatment with HDAC inhibitors (HDACi) prevented excessive proliferation in human melanoma cells, suggesting functional roles of observed chromatin state transitions in driving hyper-proliferative phenotype 44.

In this context, the locus *INK4a-ARF,* which plays key role in melanomagenesis 45, is subjected to histone acetylation modifications leading to the deregulation of the cognate tumor suppressor genes expression *p14^ARF^* and *p16^INK4A^* 46,47. With the emergence of a role for HDACs in melanoma pathogenesis and the increasing availability of HDACi being developed, epigenetic-targeted therapies have gained some attention. Several groups have previously reported the effect of pan‐HDACi on tumor cells in a variety of cancers 48,49. In melanoma, it has been shown that the pan‐HDACi panobinostat (LBH589) exerts a dual effect on melanoma cells by affecting growth/survival through increased apoptosis along with a G1 cell cycle arrest and by increasing melanoma immunogenicity 50. Another study showed that the combination of panobinostat with BRAFi have synergistic effects on BRAFi-resistant melanoma by decreasing the PI3K pathway activity and by changing the balance between pro- and anti-apoptotic proteins 51. Vorinostat, another HDACi, is known to induce ROS 52. Since elevated levels of ROS are found in drug-resistant cells, vorinostat was used to further increase these ROS levels to trigger apoptotic death selectively in the drug-resistant tumor cells 53. However, one of the most challenging issues with the use of HDACi is to attribute the effect to a single HDAC or to a particular sub‐group of HDACs and determine the HDAC(s) responsible for these anti‐tumor effects. To address this question, Waon *et al.,* evaluated the effect of various pan‐ and selective-HDACi on a broad panel of human melanoma cell lines. They assessed effects of pan-HDACi (LBH589 and TSA), class I and IV inhibitor (MGCD0103), or the HDAC6 inhibitors (Tubastatin A 54 and Nexturastat A 55). Interestingly, selectively inhibiting HDAC6 recapitulated the anti-proliferative effects of pan-HDACi 56. Further, the same group showed that HDAC6 is an important regulator of the JAK/STAT3 pathway *via* production of IL6 in response to LPS 57 and in turn PD-L1 expression rending its selective inhibition as a potential immuno‐modulatory option in current therapies 57.

In melanoma, T-box 2 (Tbx2) downregulates expression of the cell cycle inhibitor *CDKN1A* (p21) by targeting HDAC1 to its promoter. This leads to senescence bypass and melanoma progression 58.

Wilmott *et al.,* have shown that increased percentage of nuclear HDAC3 and cytoplasmic HDAC8 is associated with better prognosis from the time of diagnosis of primary melanoma 59. Collectively, this suggests that nuclear expression of some HDAC, such as HDAC3, are good prognostic factors and that some HDAC such as HDAC8 could exert good prognosis when having cytoplasmic functions beyond their classical role. However, a recent study showed that multiple stress exposure on melanomas such as BRAFi and MEKi combination, increased HDAC8 expression and lead to a drug-resistant phenotype 60. The authors showed that HDAC8 is implicated in MAPK and AP-1 signaling regulation by deacetylation of c-Jun increasing its transcriptional activity. Importantly, xenograft studies supported a critical role for HDAC8 in therapeutic response upon non-selective (panobinostat) or HDAC8 specific inhibitor (PCI-34051) treatment by increasing targeted therapy durability 60. Moreover, additional studies described HDACs not only as histone modifiers but also to have the capacity to modify other proteins unrelated to the chromatin environment 61,62.

Noteworthy, most of the US Food and Drug Administration (FDA)-approved HDACi showed significant CR for the treatment of lymphomas, Cutaneous T-cell lymphoma (CTCL) and Peripheral T-cell lymphoma (PTCL). Unfortunately, HDACi monotherapy has not demonstrated similar success in solid tumors. The poor efficacy of HDACi in solid tumors compared to hematological malignancies is still poorly understood. One possibility could be that HDACi reach their therapeutic concentrations more efficiently in hematological malignancies so that the short-life may not affect their activities as it could be the case in solid tumors. Moreover, in phase II clinical trials with HDACi as monotherapies against solid tumors, only a small subset of patients presented CR or partial response along with severe adverse effects 63. A Phase II clinical trial with entinostat (inhibitor of class I HDAC enzymes) for patients with metastatic melanoma pretreated with systemic therapies (at least one and no more than two) reported adverse events (e.g. nausea, hypophosphatemia, pain in extremeties, diarrhea) 64. Recently, Maertens *et al*., highlighted the use of HDACi to potentiate MAPKi effects in melanoma 65. They have shown that genetic or chemical suppression of HDAC3 using entinostat potently cooperates with the combination dabrafenib/trametinib in *BRAF-*mutant melanoma and in difficult-to-treat *NRAS-* and *NF1-*mutant tumors. Moreover, they found that *MGMT* expression serves as a biomarker for this triple BRAF/MEK/HDAC inhibitor combination efficacy. Mechanistically, this combination triggers severe DNA repair defects by suppressing the expression of ELK which regulates key genes involved in this process (e.g. *BRIP1*, *PARP3*, *XRCC5*) ultimately leading to enhanced melanoma cells death 65.

Of note, deregulation of histone demethylases resulting in aberrant histone methylation patterns have been linked to melanoma pathogenesis (**Figure 3**). Using the H3K4me3 demethylase JARID1B (i.e KDM5B) as a biomarker, Roesch *et al.,* characterized the existence of a slow cycling subpopulation of cells, within the rapidly proliferating main population 66. The slow-cycling JARID1B-positive subpopulation shows increased *in vitro* self-renewal and knockdown of JARID1B caused exhaustion of melanoma cells 66. Collectively, JARID1B-positive cells are critical for continuous melanoma tumor growth.

This subpopulation was found to be highly dynamic underlying the variable nature of the epigenetic landscape of melanoma 66. Importantly, the discovery of this slow-cycling subpopulation was of critical clinical importance considering its role in melanoma maintenance since the majority of current therapies target proliferating cells. Further, characterization of the slow-cycling JARID1B (high) phenotype revealed a high expression of mitochondrial bioenergetic enzymes and blocking the mitochondrial respiratory chain overcomes intrinsic multidrug resistance in melanoma 67.

Moreover, it has been shown that two different types of histone H3 lysine 9 demethylases, Lysine-Specific Histone Demethylase 1A (LSD1 i.e KDM1A) and Jumonji Domain-Containing Protein 2D (JMJD2C i.e KDM4C), promote the bypass of oncogenic HRas^G12V^- or Braf^V600E^-induced senescence by preventing H3K9 Trimethylation at E2F target gene promoters, thereby favoring melanomagenesis. Inhibition of these H3K9 demethylases restored senescence and growth arrest 68. Interestingly, a recent study described an effective dual pharmacological inhibitor of the CoREST complex containing HDAC1 along with LSD1 in slowing tumor growth 69. However, the regulation of methylation is complex since histone hypermethylation induced by low glutamine in tumor core regions or in patient-derived BRAF^V600E^ melanoma cells resulted in cancer cell de-differentiation or resistance to targeted therapy which will be discussed later. Importantly, knockdown of the H3K27-specific demethylase KDM6B (i.e jumonji domain-containing 3, JMJD3) reproduced the low-glutamine effects *in vitro* and *in vivo*, whereas EZH2 knockdown (described in the “writers”) attenuates them 70. Another study also involved KDM6B in melanoma pathogenesis. Indeed, the authors identified a novel epigenetic mechanism by which KDM6B transcriptionally upregulates several targets of NF-κB and BMP (Bone Morphogenic Protein) signaling to promote melanoma progression and metastasis 71.

Importantly, some of these studies suggested their respective histone demethylases or deacetylases as a potential target for melanoma treatment supporting the reversible aspect to explore previously mentioned (**Table 1** for selective inhibitors).

### Chromatin remodeling complexes

Importantly, SWI/SNF member's alterations have been linked to melanoma. Especially, loss-of-function mutation in components of this complex such as ARID2, ARID1A, ARID1B or SMARCA4 are found in 13% of melanomas, suggesting a tumor suppressor role and highlighting the importance of chromatin remodeling in melanomagenesis 72,73. Interestingly, at least one of the ATPase subunits BRG1 or BRM is required for melanoma tumorigenicity and most likely promote expression of distinct target genes 74. It has been shown that BRG1 takes part in a novel form of the PBAF chromatin remodeling complex along with CHD7, and interacts with the Microphthalmia-associated transcription factor (MITF) 75. The authors showed that MITF and SOX10 actively recruit BRG1 to a set of MITF-associated regulatory elements (MAREs) at active enhancers and that BRG1 also regulates the dynamics of MITF genomic occupancy. This interplay along with additional transcription factors is essential for transcription regulation and many aspects of melanocyte and melanoma cell physiology 75. In line with this study, using a mouse melanoma model conditionally expressing BRAF^V600E^ along with *Pten* inactivation that rapidly develop melanoma, it has been shown that somatic inactivation of *Brg1* and *Bptf* (the defining subunit of the NURF complex) delay tumor formation and deregulate a substantial and common gene expression programs critical for normal tumor cell growth. These two subunits also coregulate with *Mitf* and *Sox10* many genes supporting a cooperation between transcription factors and chromatin remodeling complexes to dictate fundamental gene expression programs in melanoma 76. The same group also reported that the NURF complex interacts with MITF and uncovers a role for the defining subunit of this complex, BPTF. The study shows that *Bptf* regulates proliferation, migration and morphology of murine melanoblasts *in vivo* and is essential for differentiation of adult melanocyte stem cells 77. These studies are of critical importance since MITF is a key driver of plasticity 78,79, allowing the transition of melanoma cells between a differentiated-proliferative phenotype and a stem cell like slow cycling-motile phenotype 80-82. Finally, it has been shown that ATRX loss (another SWI/SNF chromatin remodeler) correlates with melanoma progression 83.

Of note, several subunits in the SWI/SNF complex, including SMARCA4, SMARCA2, BRD9, and PBRM1 contain druggable bromodomains. Synthetic lethal interactions involving several of these subunits have opened the possibility of new therapeutic strategies 84.

### Histone variants

With different sequences and properties, histone variants replace the canonical histones into defined regions of the genome driven by histone chaperones and therefore modify chromatin structure and gene expression 12. We will discuss here few of the first discoveries including variants of H2A and H3 in melanoma pathogenesis. MacroH2A is generally considered to be transcriptionally repressive. It suppresses melanoma progression *via* transcriptional repression of CDK8, which is required for proliferation of melanoma cells 85. On the other hand, macroH2A loss directly contributes to melanoma progression by promoting tumor growth and metastatic potential 85. Overexpression of H3.3, a variant of H3, represses E2F target genes and triggers senescence 86. More recently, a role for a specific isoform of the H2A.Z variant, H2A.Z.2, has been described in melanomagenesis 87. H2A.Z.2 is highly expressed in melanoma and high levels correlate with poor patient survival. That study demonstrated that H2A.Z.2 binds and stabilizes BRD2 to promote cell cycle progression by controlling the transcriptional output of E2F target genes. Finally, H2A.Z.2 deficiency increased sensitivity of melanoma cells to chemo- and targeted therapies (MEKi) 87. The essential role of these variants in melanoma or the chaperones upstream depositing them into defined region of the genome, should be taken in consideration to explore potential therapeutic strategies to alter sensitivity of melanoma cells to current therapies.

## Epigenetic impact on targeted therapy efficiency

### MAPK pathway and targeted therapies

Hyperactivation of the MAPK signaling pathway v*ia* mutations in *BRAF*, *NRAS*, or *NF1*, drives CM progression, underlining the fundamental role of controlled MAPK signaling for melanocyte homeostasis 88-90. As such, efforts have been directed towards this pathway for targeted cancer therapies. BRAF^V600E^ is the most common mutation in CM (>50%), which leads to constitutive activation of MEK/ERK signaling independently of upstream Receptor Tyrosine Kinases (RTK) or RAS activation, resulting in recurrent positive regulation of genes involved in cell proliferation and survival 90, that is, uncontrolled cell proliferation.

BRAF inhibitors (BRAFi: vemurafenib, dabrafenib, encorazfenib) and more recently, the combination of a BRAFi and MEKi (cobimetinib, trametinib, binimetinib) have shown remarkable clinical activity in advanced metastatic CM in patients with mutant BRAF^V600E/K^ 92-95. However, their use is conditioned by the presence of the activating mutation and therefore can only benefit up to 50% of patients. Moreover, ~60% of patients with this mutation respond well to ERK signaling inhibitors, however patients almost invariably develop resistance and relapse within a 6-9 month period 88.

Mechanisms underlying acquired resistance to ERK signaling inhibitors include alterations of BRAF^V600E^ (overexpression, amplification and aberrant splicing) 96,97, upregulation of kinases (e.g. Tpl2/Cot) 98 and RTK 99 or RAS mutations 100, amongst others - all of which reactivate ERK signaling. However, these mechanisms account for a fraction of acquired resistance. Collectively, novel targets and therapies are still critically needed.

### Epigenetic mechanisms of resistance to targeted therapies

The place that epigenetics is taking in melanoma pathogenesis is undeniable over the last few years. Not only epigenetic alterations were proved to be involved in melanoma development but also in mechanisms underlying acquired resistance to targeted- and immunotherapies that we will discuss here after (**Table 2**).

In a panel of tumor biopsies that have acquired resistance to MAPK inhibitors (MAPKi), for approximately 40% of them, no validated mutational mechanism was identified 7. Recent reports have implicated DNA methylation, transcriptional changes, microRNA alterations, as well as microenvironmental stressors in promoting melanoma drug resistance to MAPKi in BRAF^V600-^mutant melanoma 7,101-104. These highly recurrent non-genetic mechanisms clearly support the necessity to dig into transcriptomic and epigenetic alterations in addition to genetic events to better grasp the burden of melanoma resistance and to develop new combinations of therapeutic strategies.

Importantly, it suggests that epigenetic alterations may play a key role in rewiring the chromatin landscape of melanoma cells to allow adaptation to current therapies. Owing to the fact that chromatin-mediated changes are reversible processes, the most clinically relevant observations implicating such regulations in drug resistance would be the “drug holiday” concept. It consists of a treatment “break” or intermittent treatment, which delays resistance whereas a genetic regulated drug resistance would not be affected. Indeed, re-challenging two patients with BRAFi after a treatment free period and disease progression upon BRAFi or BRAFi+MEKi administration resulted in a significant CR indicating that resistance to BRAF-selective inhibitors can be reversible following treatment interruption 105. Supporting that, a recent retrospective study for patients retreated with BRAF-targeted therapy after disease progression and treatment “break” showed 43% of clinically significant response 106. The emergence of clinical evidences of a reversible aspect for drug resistance highlights previous findings almost a decade ago where slow cycling subpopulations of cancer cells, including those of melanomas, have been implicated in reversible drug tolerance. However, additional mechanisms are not to be excluded such as induction of cancer cell drug addiction, matrix remodeling and secretome adaptation promoting temporary resistance to BRAFi 107-109. Using anti-cancer agents in several tumor cell lines, Sharma *et al.,* consistently identified a small fraction of cells surviving treatment with drug concentration 100-fold higher than the IC_50_ 110. This study was one of the first shedding light on such a drug-tolerant subpopulation. Importantly, a “drug holiday” period re-sensitized this subpopulation to the initial treatment. Briefly, this drug-tolerant cells displayed elevated expression of the histone demethylase KDM5A (JARID1A) and consequent reduced level of its target, the histone modifications H3K4me3/2, therefore altering their chromatin state. Finally, RNAi-mediated knockdown of KDM5A confirmed that this histone demethylase was important for the establishment of the reversible drug-tolerant state 110. Cells displaying elevated expression of another H3K4 demethylase, KDM5B (JARID1B), were also found to be enriched upon BRAFi treatment that has been linked to this drug-tolerant state phenotype as well. Importantly, inhibition of mitochondrial respiration blocks the emergence of the KDM5B(high) subpopulation and sensitized melanoma cells to therapy 67. Pushing this concept further, another study showed that chronic exposure to external stressors such as hypoxia, nutrient starvation and drug treatment give rise to an induced drug-tolerant cells (IDTCs) rather than a selection of a pre-existing subpopulation 110. In this study, microarray analyses from IDTCs cells revealed elevated expression levels for the H3K4 demethylases *KDM1B*, *KDM5A*, *KDM5B* and for the H3K27 specific demethylases *KDM6A*, *KDM6B.* A concomitant chromatin modification state was observed with a decrease of the histone marks targeted by these enzymes, respectively H3K4me3 and H3K27me3, highlighting epigenetic remodeling. On that note, loss of differentiation markers such as melan-A and tyrosinase which are MITF target genes, was also observed in IDTCs suggesting the transition into an undifferentiated state in accordance with increased aggressiveness 111. Recently, another study highlighted tumor heterogeneity as a major challenge for cancer treatment. In mouse melanomas, CD34^+^ and CD34^-^ tumor subpopulations have been characterized as melanoma-propagating cells exhibiting some key properties from stem cell or progenitor cell. Moreover, differences in tumorigenic properties, heterogeneity recapitulation and resistance have been observed in these two subpopulations 112. The authors demonstrate that CD34^+^ and CD34^-^ subpopulations harboring the BRAF^V600E^ mutation have differential response to targeted BRAFi. Linking epigenetics to tumor heterogeneity, upon exposure to targeted therapies, elevated KDM5B expression shifts melanoma cells to a more drug-tolerant CD34^-^ state while KDM5B loss shifts melanoma cells to a more sensitive CD34^+^ state. Together, these studies support a critical role for KDM5B in epigenetic regulation to orchestrate the transition of subpopulations with distinct drug sensitivity.

Furthermore, cancer cell dedifferentiation and histone methylation were attributed to resistance following BRAF inhibitor treatment 70. In that case however, the authors described a role for histone hypermethylation (H3K27me3) in tumor core regions specifically, that resulted in cancer cell dedifferentiation and resistance to BRAF inhibitor treatment 70. The variability of these studies regarding the role of H3K27me3 in resistance to BRAFi could be explained by the difference in the models used (e.g. melanoma cell lines and tumors). Nevertheless, these studies involved chromatin remodeling such as loss or gain of histones post-translational modifications impacting melanoma cells response to external stressors (hypoxia, drug exposure, low nutrients) and highlight the importance of the microenvironment impact onto epigenetic alterations, intra-tumoral heterogeneity and the therapeutic response.

The emergence of new technologies over the last years (e.g. single cell analysis or genome editing using the Clustered Regularly Interspaced Short Palindromic Repeats (CRISPR)-associated protein 9 (Cas9) system) have become attractive and powerful tools for biological discoveries and the identification of novel drug targets. Analyses at the single cell level of human melanoma cells allowed Shaffer *et al.,* to identify a transcriptional variability in which the cells with the capability to resist drug treatment can be predicted 113. Expression of few resistance markers are found at high levels in a small number of cells and the addition of drug triggered an epigenetic reprogramming to switch from a transient transcriptional state into a stably resistant state. Especially, this transition is mediated first by a dedifferentiation state followed by activation of new signaling pathways mediated respectively by loss of SOX10 (regulates neural crest development in melanocytes) and activation of TEAD (regulates invasion in melanoma) among others 113.

Taking advantage of the simplicity of programming the CRISPR-Cas9 to modify specific genomic loci, Shalem *et al.,* interrogated on a genome-wide scale, gene function in melanoma resistance by screening for genes whose loss is involved in resistance to vemurafenib 114. This study identified members of the STAGA HAT complex (e.g. TADA2B, TADA1), consistent with a critical role for histone acetylation in melanoma drug resistance 114. Taking all this in consideration, there is no doubt today that shedding light onto the transcriptomic and epigenetic alterations underlying acquired MAPKi resistance in melanoma is of critical importance to improve patients' clinical outcome.

Moreover, several studies highlighted a role in melanoma resistance for sirtuin proteins constituting the class III HDACs. Of note, SIRT1, 2, and 6 are considered the nuclear SIRTUINs and are chromatin-bound 115. Previous report on sirtuins in melanoma showed that SIRT1 inhibition decreases melanoma cell growth and rescues the sensitivity to PLX4032 of PLX4032-resistant BRAF^V600E^-mutated melanoma cells 116. On the other hand, an shRNA screen identified that SIRT2 depletion conferred resistance to MAPKi in BRAF^V600E^ melanoma cells through ERK reactivation 117. Interestingly, the role of SIRT6 in the pathogenesis of several cancers has been controversial. Indeed, in the last years, SIRT6 has been described either as a tumor suppressor or an oncogene in tumorigenesis regulation through diverse biological pathways 118. This pleiotropism, that can be extended to the Sirtuin family, adds a layer of difficulty in studying the cellular mechanisms by which sirtuins impact cancer or biological processes but makes it extremely exciting.

We favored the hypothesis that epigenetic mechanisms altering gene expression programs contribute to ERK signaling inhibitor resistance. Using a CRISPR-Cas9 screen approach focused on chromatin factors to identify epigenetic players in melanoma drug resistance, we identified the chromatin associated histone deacetylase SIRT6 as a regulator of resistance to the clinically relevant BRAFi (dabrafenib) and BRAFi+MEKi (dabrafenib+trametinib) combination 119. Interestingly, we have also identified the histone acetyltransferases (HATs) KAT1 (HAT1) and KAT2B (PCAF), again supporting the importance of the reversible aspect in chromatin mediated processes and the balance of opposing factors in orchestrating the chromatin state in diseases. However, the role of KAT1 and KAT2B remain to be explored in melanoma drug resistance.

In our study, we uncovered a new role for the NAD-dependent chromatin-associated deacetylase SIRT6 in melanoma drug resistance 119. Using, a combination of transcriptomic, epigenomic and proteomic analyses we demonstrated that haploinsufficiency of SIRT6 in BRAF-mutant melanoma cells decreases sensitivity to MAPKi independently of the ERK signaling pathway. This allows cells to survive by increasing their IGFBP2 expression which in turn activates their IGF-1R receptor and downstream AKT survival signaling in presence of these inhibitors 119. Consistent with our results, a link between the insulin/IGF and sirtuin pathways has been reported previously in the development of cardiac hypertrophy and heart failure 120. On the other hand, previous studies have also suggested that increased activation of the AKT signaling pathway plays a role in MAPKi resistance 99,121,122. Importantly, a recent study identified *IGFBP2* as part of a gene signature in response to MAPKi “drug-tolerant persisters” 102 and there is little evidence for its use as a biomarker. Strikingly, we observed that IGFBP2 protein levels correlated with resistance to MAPKi in several BRAF-mutant melanoma cell lines and are associated with poor prognosis in primary melanomas. Importantly, we showed that co-targeting the MAPK and IGF-1R pathways can prevent/delay resistance to targeted MAPKi therapies, particularly for patients with high levels of IGFBP2, highlighting the importance of early detection as previously mentioned.

Intriguingly, we also observed that melanoma cells devoid of SIRT6 undergo chromatin reorganization reflected by increased open chromatin and H3K56ac at these sites 119. Such potential for genomic instability is consistent with increased DNA damage and impaired tumor growth such as previously reported in acute myeloid leukemia (AML) 123. Together, these data suggest additional functions for SIRT6 to explore in melanoma biology, in which we could consider SIRT6 “complete” depletion as a novel strategy in melanoma pathogenesis to enhance their sensitivity to current targeted MAPK therapies. Our study and others, highlight here the importance of the epigenetic balance and how the levels of chromatin factors can be critical in disease biology, in our case, with two opposite outcome observed for SIRT6 haploinsufficiency versus SIRT6 deficiency 119.

While we can refer to these cells as “IDTCs; drug tolerant persisters or slow cycling drug tolerant cells”, we now know that the burden of acquired melanoma resistance not only arise from genetic alterations but also from the emergence of these subpopulations. Considering that acquired drug resistance may involve multiple distinct molecular mechanisms taking place independently within the same patient, strategies to overcome such resistance with a single targeted agent remain extremely challenging. Therefore, early treatment to short-circuit this drug tolerant state to prevent or delay drug resistance is particularly attractive (**Table 2**).

## Epigenetic impact on immunotherapy efficiency

### Immune regulation and checkpoint inhibitors therapies

The second therapeutic revolution in melanoma came from a deeper understanding of the tumor microenvironment and immunophenotype of tumors. Melanomas are not isolated entities but are dependent on their surrounding cells whom they are in constant dialogue. These include fibroblasts, immune cells, and endothelial cells that constitute the tumor stroma and that allow tumor cells to adapt to their changing microenvironment and therefore survive and replicate 124. Stroma composition is heterogeneous and dynamic. The immune system is also very heterogeneous in itself and can lead to a state of immunosurveillance allowing efficient tumor elimination or a state of immunotolerance promoting tumor escape and therefore tumor survival. Thus, the challenge of effective immunotherapy is to not only directly promote tumor death but importantly, to modulate its microenvironment in order to obtain a state of immunosurveillance 125.

In this regard, one of the great breakthroughs in medicine over the last 10-15 years have been the discovery of how the immune system fights against cancer thanks to the work by Allison and Honio whose Nobel-winning research led to the development of immune checkpoint inhibitors (ICI). ICI is a form of cancer immunotherapy which targets immune checkpoints, that is, immune cells which dampen the immune response following an immunologic stimulus. The rationale behind this strategy is to stimulate patient's own immune system to recognize and destroy their cancer cells more effectively. This is achieved by blocking checkpoint proteins such as programmed cell-death protein 1 (PD-1) and cytotoxic T-lymphocyte-associated antigen 4 (CTLA-4) which are inhibitory receptors expressed normally on activated effector T lymphocytes which act as 'brakes' to turn off T-cell responses and prevent T cells attacking their own cells. By avoiding detection, malignant cells are able to spread uninhibited. The ligand for PD-1 (PD-L1) is strongly expressed by melanoma cells. Immunotherapy aims to block the interaction between PD-1 on T cells and its ligand PD-L1 on tumor cell or alternatively, blocking the interaction between CTLA-4 on T cells and its co-stimulatory receptors CD80, CD88 or B7 on antigen presenting cells. The use of anti-CTLA-4 antibody with Ipilimumab (now also with Tremelimumab) was the first immune checkpoint inhibitor drug that prolonged overall survival in patients with advanced cutaneous melanoma 126. Later, anti-PD-1 (Nivolumab, Pembrolizumab and most recently Cemiplimab) and anti-PD-L1 (Atezolizumab and Avelubam) antibodies have similarly shown improved survival and progression-free survival in these patients 126. The IFN-γ, JAK/STAT pathway appears critical for the response to immune checkpoint blockers 127*.*

Although ICIs have revolutionized the treatment of patients with advanced melanoma, approximately one third of patients benefit from anti-PD1 treatment but responses are often associated with immune-related adverse events (irAE) which can be serious and life-threatening 128. Albeit such progress, the alarming trend is that the global incidence of cutaneous melanoma has been steadily increasing over the last 60 years with two thirds of all patients with advanced forms of the disease who still fail to get a long-term benefit of the treatments. This area of research clearly warrants urgent attention to identify potential new therapeutic targets.

One of the pathways which clearly needs fine-tuning is the more specific recognition of neoplastic cells to reduce the incidence of irAE. In this regard, the innate immune system which relies on the potent and critical anti-tumor function of natural killer (NK) cells acting in concert with surrounding dendritic cells and macrophages to destroy the tumor is likely to be an attractive target. The prompt response of NKs is due to their release of pre-formed cytotoxic mediators and expression of surface ligands that are able to trigger death receptors on target cells (innate response), as well as their ability to produce a wide variety of chemokines and cytokines to recruit and instruct other immune cells for subsequent priming (adaptive response). In this regard, the latest paradigm shift which demonstrates innate immune cells to have memory properties may offer a lot of promise 129. The mechanisms behind this trained immunity include changes in intracellular metabolism and epigenetic regulation at the level of histone modification 130,131. Epigenetic remodeling is a common feature of human melanoma and other tumor types and plays a key role in the immune escape of neoplastic cells from antigen specific T cell recognition. Epigenetic drugs have been demonstrated to improve recognition of cancer cells, to have strong immunomodulatory activity and to be able to reverse epigenetically driven immune alterations suggesting a combination of agents that target both epigenetic and immunotherapy approaches may improve the efficacy and specificities of treatment against tumor 132.

### Epigenetic mechanisms of resistance to immunotherapies

Unfortunately, as for any therapies in melanoma, resistance ultimately develops. Despite recent studies pointing out epigenetic regulations contributing to tumor immune escape, antigen expression or presentation, regulating tumor cell killing or T-cell response (and we refer the reader this review for more details 133) our knowledge in epigenetic mechanisms involved in resistance to immunotherapies is still in its infancy. Indeed, to the best of our knowledge, only a few studies to date reported epigenetic players in melanoma immunotherapy resistance (**Table 2**). For instance, Zingg *et al.*, described a role for EZH2 in acquired resistance to cancer immunotherapy 134. In particular, they showed that EZH2 is upregulated upon anti-CTLA-4 or IL-2 immunotherapies in cancer cells, leading to a loss of tumor control. Mechanistically, activation of EZH2 promotes H3K27 trimethylation and consequent suppression of essential immune-related genes 134. Importantly, GSK503 an inhibitor of the methyltransferase activity of EZH2, restored tumor immunogenicity and T-cell infiltration and suppressed melanoma growth upon immunotherapy. In that study, Zingg *et al*., provided an insight into the effects of EZH2 activation which resulted in adaptive cancer resistance to immunotherapy and they provided a rationale for combinatorial epigenetic immunotherapy approach. Using a genome-scale CRISPR-Cas9 screen, a recent study identified key chromatin regulators of tumor immune resistance 135. Briefly, inactivation of either ARID2, PBRM1 and BRD7 of the PBAF complex sensitized melanoma cells to cytotoxic T-cells *via* an enhanced response to IFN-γ 135. Of note, PBAF-deficient tumor cell lines produced higher amounts of chemokines, therefore allowing more efficient T-cell infiltration into the tumor site. Another study implicated the histone H3K4 demethylase LSD1 in resistance to anti-PD1 therapy 136. The authors showed that double-stranded RNA (dsRNA) stress, resulting from LSD1 loss, led to potent anti-tumor T cell immunity. Importantly, LSD1 depletion renders refractory mouse tumors responsive to anti-PD-1 therapy 136. These studies highlight the role of chromatin regulators whereby their inhibition reverses certain features of adaptive resistance of tumors to immunotherapy, supporting again the importance of the reversible aspect of epigenetic processes (**Table 2**). Moreover, this provides a strong rationale for implementing epigenetically-based immunotherapies in cancer patients.

Therapies targeting anti-tumor T-cell responses were successful in a variety of diseases, unfortunately, most patients still do not respond, underlying a critical need for approaches which improve immunotherapeutic efficacy. For instance, combination of HDACi with immunotherapy (anti-Pmel T-cell transfer plus Pmel peptide-pulsed DC vaccine) decreased tumor volume by 70% compared to untreated mice, where reductions in tumor volumes of 49% and 21%, were achieved with immunotherapy or HDACi alone, respectively 137. The efficacy of combining epigenetic modulators such as the DNA hypomethylating agent 5-aza-2'-deoxycytidine (5-AZA-CdR) and immunotherapy caused a 77-81% reduction in tumor volume and was much more effective than the effect mediated by single agents 138. On the same note, a recent study from Laino *et al*., highlighted a role for HDAC6 on immune function of melanoma patient T-cells 139. Using the HDAC6-specific inhibitors, ACY-1215 and ACY-241, on T-cells from metastatic melanoma patients, the authors observed decreased cytokine production (IL-4, IL-5 and IL-6), decreased FOXP3 expression (a master regulator of regulatory T cells) and higher T-cell infiltration in melanoma upon treatment 139. This study demonstrated that the use of HDAC6-specific inhibitors decreases immunosuppression and enhances immune function of melanoma patient T-cell giving a rationale for a potential translation into clinic. Taking all this in consideration, efforts taken in studying the different mechanisms of intrinsic and extrinsic resistance post immunotherapy need to be intensified to improve combinatorial epigenetic immunotherapy approaches. A summary of epigenetic drugs in combination with immunotherapy in ongoing clinical trials for the treatment of melanomas is discussed in Table 3.

## Epigenetics in uveal melanomas (UM)

While most melanomas do form on the skin, it can also arise in the eye, known as ocular melanoma. Large majority of ocular melanomas originate from uvea (95%), involving the posterior uvea (choroid 90% and the ciliary body 5%) and anterior uvea (iris 5%). Uveal melanoma (UM) is the most common primary cancer of the eye in adults. In U.S. and Europe, UM has an incidence of 5 cases per million people per year. Despite successful treatment of the primary lesion, liver metastases develop in half of these patients 140. The etiopathogenesis and biological behaviors of UM are very different from cutaneous melanoma 141. They display distinct landscapes of genetic alterations with different metastatic routes and tropisms. Hence, therapeutic improvements achieved in the last few years for the treatment of CM have failed to improve the clinical outcomes of these patients.

Genetic alterations most often observed in UM are somatic activating mutations in the G-protein coupled receptor GNAQ signaling cascade 142-145 associated with mutations prognostically significant of the metastatic risk in *BAP1*, *SF3B1*, and *EIF1AX* (BSE mutations) 146. In addition, copy-number variations can also be detected in the context of the BSE mutational status and specific gene expression signature. Collectively, these different alterations predict UM subtypes. Currently, there are no approved systemic treatments for UM once it has spread 147. 90% of patients will die within 6 months after diagnosis of metastases (review 141). Thus, this is really an area of urgent need for research to find more efficient treatments for UM.

Changes in the epigenetic landscape, including DNA methylation, histone modification and small non-coding RNA have also been reported in UM. Given the reversible nature of some epigenetic regulations, inhibition of the epigenetic enzymes in cancer cells might switch these modifications back to a “normal-like” chromatin landscape. As for CM described above, only histone modifications will be addressed in detail.

As mentioned above, one of the most prominent alterations found in UM is the loss of the tumor suppressor BRCA-1 associated protein-1 (*BAP1*) gene. BAP1 has one copy that is often lost *via* monosomy of chromosome 3 and the second copy by mutation. BAP1 is the catalytic subunit of the PR-DUB complex that deubiquitinates histone H2A 148. Consistently, knockdown of BAP1 in UM cells induced a marked increase in H2A ubiquitination 149. Ubiquitinated H2A is the most prevalent ubiquitin conjugate in cells which is linked to the Polycomb protein complex 1 (PRC1) ubiquitin ligase activity 150. In the nucleosomes, ubiquitinated H2A is situated close to linker histone H1. Deubiquitination of H2A initiates transcriptional activation *via* linker histone H1 dissociation 151 and *via* trans-histone cross-talk with H3K4 di- and trimethylation 152.

Depletion of BAP1 in cultured cells induces a switch in transcriptional programs from differentiated poorly aggressive Class 1 to dedifferentiated highly aggressive Class 2 gene expression profile and re-programmation of UM cells towards a stem-like phenotype 149,153. The stem-like phenotype is associated with quiescence and motile ability, thereby suggesting that the loss of BAP1 may be mechanistically linked to the metastatic ability. A recent study from Field *et al*., went further on characterizing the impact of *BAP1* loss on DNA methylation in UM 154. Here, the authors analyzed global DNA methylation in 47 Class 1 and 45 Class 2 primary UMs and in engineered UM cells where BAP1 was inducibly depleted. Moreover, they analyzed RNA-seq data from 80 UM samples and engineered UM cells. They observed hypermethylation on chromosome 3 coupled with decreased gene expression at several loci among which, *BAP1* is located. The deregulated genes identified are involved in axon guidance and melanogenesis with many located on chromosome 3 (e.g. *MITF*, *SATB1*, *ROBO1* or *SEMA3B*). Interestingly, *BAP1* itself might be epigenetically regulated since a hypermethylated site was identified in the *BAP1* locus for all the class 2 tumors. By inducibly knocking down *BAP1* expression, a methylomic repatterning was observed and enriched for genes similar to UM tumors. This study supports previous work and suggests a chronological order for UM divergence from Class 1 to Class 2 with loss of one chromosome 3 copy, a *BAP1* mutation on the other copy leading to a methylomic redistribution characteristic of Class 2 UMs, thereby a more aggressive state 154.

Meantime, HDAC inhibitors are reported to decrease histone H2A ubiquitination through transcriptional repression of the PRC1 component BMI1 155. Hence, HDACi emerged as promising drugs in the treatment of UM to fight the H2A hyperubiquitination phenotype caused by *BAP1*-deficiency. HDAC inhibitors such as the pan-HDACi suberoylanilide hydroxamic acid (SAHA, Vorinostat) and class I-selective HDACi Romidepsin (FK-228) have been FDA-approved to treat patients with cutaneous T cell lymphoma and are well tolerated 156,157.

In the context of UM, HDACi including valproic acid (VPA), trichostatin A (TSA), LBH-589, and SAHA have been assessed. They reverse the H2A hyperubiquitination caused by BAP1 loss and convert highly aggressive UM cells to a low-grade, differentiated state 149. A phase 1 clinical trial is assessing the ability of vorinostat to induce the switch of class 2 UM cells into a cell phenotype that resembles normal melanocytes (NCT03022565).

VPA, LBH-589, TSA and SAHA have been described to inhibit proliferation *in vitro*, yet they did not induce much cell death. VPA has been also reported to inhibit UM tumor growth *in vivo* 149. Of note, BAP1-deficient UM cells seem more sensitive to HDACi than BAP1-proficient cells 149. A phase 2 clinical trial is testing the effect of VPA on tumor growth in Class 2 metastatic UM patients (NCT01587352).

Other HDACi including JSL-1 158, quisinostat 159 and the Sirtuin 1-2 inhibitor Tenovin-6 160 have been shown to induce apoptosis *in vitro*. However, it appears that HDACi possess poor anti-cancer clinical activity against solid tumors when used as a monotherapy.

Moreover, the clinical use of MEKi in UM is limited since acquisition of resistance has been observed along with adverse effects 161,162. Thus, combining epigenetic drugs and chemotherapy, immunotherapy or targeted therapy may prove to have clinical value especially in the case of UM where loss of *BAP1* and epigenetic alterations are critically involved in the pathogenesis. Several lines of evidence indicate that the combined therapy could be promising. Using multiomics approaches and drug screens, a recent study identified the pan-HDACi panobinostat to restrain MEKi resistance 163. The authors identified several potential pathways to target that were upregulated upon MEKi including the PI3K/AKT, ROR1/2 and IGF-1R signaling pathways. They also observed increased GPCR expression leading to therapeutic escape through YAP signaling. Finally, their screen compounds identified panobinostat as a potential inhibitor to suppress YAP and AKT signaling activation upon MEKi that was validated *in vivo* with a long-term decrease of tumor growth 163. This study provides a rationale for the use of HDACi in combination with MEKi in patients with advanced UM. Combination of quisinostat and pan-CDK inhibitor flavopiridol 159 as well as the combination of Tenovin-6 and vinblastine 160 are synergistic in inducing apoptosis of several UM cell lines. Interestingly, Tenovin-6 purges cancer stem cells 160.

Further, the class I-specific HDACi MS-275 (Entinostat) can synergize with the pro-apoptotic ligand of the TNF family TRAIL to promote apoptosis of UM cells. MS-275 increases in a variable manner expression of the TRAIL receptors DR4, DR5, and procaspase 8 as well as recurrently inhibits expression of the anti-apoptotic effector cFLIP expression 164,165.

A phase 2 clinical trial, evaluating the efficacy of concomitant use of pembrolizumab and entinostat in UM (NCT02697630) has been launched.

*Bap1* has also been shown to alter other histone marks. In a model of hematopoietic transformation in mice,* Bap1* loss is associated with decreased H4K20me1 at the *EZH2* locus, allowing its expression and in turn catalyzes H3K27me3 166. Regulation of H4K20me1 is mediated through SETD8 the only known methyltransferase that places H4K20me1 on chromatin 167.

Further, the myeloproliferation syndrome associated with *Bap1*-KO mice is reduced by treatment with the small-molecule EZH2 inhibitor EPZ011989, suggesting that EZH2 might represent a therapeutic target in *BAP1*-deficient malignancies, including UM cells.

However, targeting EZH2 might not have clinical value in UM for several reasons. Indeed, the expression of *EZH2* appears similar in *BAP1*-deficient or proficient-UM cells regardless of the *BAP1* mutational status or protein expression levels. Moreover, UM cell line are not sensitive to the EZH2i EPZ-6438 168. Thus, the effect of EZH2 might be context dependent. Considering the lack of treatments in UM there is an urgent need for research and for efficient therapeutics to defeat metastatic UM and improve patient survival.

## Epigenetic modifications and therapeutics

The significant role that is taking epigenetic dysregulation in melanoma inspires scientists to orientate their studies into compounds that target epigenetic regulators. As described in this review, there are three main categories: “writers, readers and erasers”. Few drugs inhibiting epigenetic writers and erasers have been FDA-approved for the treatment of cancer. To the best of our knowledge, the only compounds targeting histone function approved in the clinic for melanomas patients are HDACi. However, the studies discussed in this review raise several points and highlight important aspect in using HDACi as potential combined anti-melanoma options. First, would it not be better to focus our effort in developing selective HDACi and to better understand their biological functions to reduce unwanted side effect observed with pan‐HDACi in clinical trials 49? On that note, although reversing gene expression repression remains attractive, a major issue to date in the use of HDACi lies on their capacity to alter epigenetic process for a specific subset of genes or cell type. For instance, the increase in acetylated histones upon such treatments could impact previously silenced tumor suppressor genes as much as oncogenes. We also need to take into consideration how this epigenetic landscape rewiring impacts normal cells? A possibility could be a malignant transformation leading to carcinogenesis due to genomic instability upon treatment. Moreover, HDACs have a variety of functions and display different expression profiles in normal cells, the effects of HDACi in cancer cells will most likely be tissue-dependent 169, underlining the importance of validating targets in a tissue-specific manner prior to treatment. Another question is whether their inhibition would benefit patients with melanoma? This is highly relevant since some HDAC can exert good prognosis when functioning in cell cytoplasm.

As previously discussed, another issue for the use of HDACi in melanoma patients is their poor efficacy in solid tumors. Further studies are essential to determine the additional functions of HDACs unrelated to the chromatin state modeling. Therefore, while preliminary results for a clinical use of HDACi are encouraging, it still has to be taken with a “grain of salt” for the reasons mentioned above. However, taking into consideration all the studies discussed in this review, it is undeniable that targeting general epigenetic players and/or transcriptional regulators would significantly potentiate anti-tumor effects although low toxicity level for normal tissues remains challenging.

## Conclusion

Several lines of evidence demonstrate that epigenetic modifications play an important role in melanoma initiation, progression, and metastasis. Along with the accumulation of knowledge on the multiplicity of epigenetic alterations that impact melanoma due to their reversible nature, here we highlight the latest research which is still in its infancy with the new but promising molecules in pipeline for future possible treatment of the disease (**Table 1-3**). Targeting epigenetic modifications is of intense interest in the treatment against cancer and epigenetic drug discovery is a rapidly advancing field. To date, these drugs have few limitations including substrate specificity, therefore many challenges remain to be resolved.

Our next challenge is to better understand the mechanisms at the origin of aberrant epigenetics that would help to identify new relevant therapeutic targets. So far, the only approved epigenetic drugs are HDACi and DNA methyl transferase (DNMT) inhibitors. Unfortunately, HDACi monotherapy has been shown to have limited efficacy against solid tumors. However, they can function synergistically with a variety of compounds or immune therapies, some of which are currently in clinical trials. Over the last few years, HMTs have been particularly attractive (e.g. SETDB1, EZH2) in the light of the structural and mechanistic data suggesting a potential modulation by small compounds. A success in one of these clinical trials (**Table 3**) would offer new hope for melanoma patients for whom other treatments have previously failed.

In the interim, histone modification signatures may guide prognosis by predicting treatment outcome and thus may support clinical decision-making in treatment of melanoma patients. We focused in this review on histone modifications among various other mechanisms, but we have to keep in mind that they are working in concert with other epigenetic aberrations such as DNA methylation or miRNA dysregulations and genetic alterations. In particular, aberrant DNA methylation associated with transcriptional repression is a major phenomenon observed in cancers. Although the exact sequence of events between histone modifications and DNA methylation remains unclear to date, these events likely drive epigenetic mechanisms together leading to malignant transformation. The classical view is that histone modifications might be the first step of epigenetic silencing, which orchestrates the recruitment of DNA methylation machinery. In that case, aberrant DNA methylation potentiate the transcriptional silencing already existing such as a lock-off mechanism. Another view is that aberrant DNA methylation has a key role in the reversion of the epigenetic state at specific genomic loci and activates silent genes. Regardless the sequence of these events, a dual inhibition using HDACi with DNMTi remains an attractive area of interest in the clinic.

It is undeniable that personalized treatments based onto the genetic and epigenetic characteristics have to be considered. Combinations of epigenetic drugs with other anti-cancer agents such as targeted therapy or immunomodulatory drugs is a promising avenue for improving the effectiveness of treatments in both cutaneous and uveal melanoma.

## Figures and Tables

**Table 1:** Epigenetic players and their impact on melanoma

Categories	Players	Target / Site	Impact on melanoma	Selective inhibitors in melanoma	References
***Writers:***	SETDB1	methylation of H3K9	favors melanoma development	CAS 935693-62-2 29	28, 29
	EZH2	methylation of H3K27	favors melanoma progression -associated with poor survival -senescence bypass	GSK503 31 EZH2i 35	30, 31, 32, 33 34
***Readers:***	BRD2, BRD4	acetylated histones	essential for tumor maintenance and melanoma cell survival	JQ1 I-BET151	38, 39, 40, 41, 42
***Erasers:***	HDAC6	regulates JAK/STAT3 and PD-L1 expression	role in immunosurveillance	Tubastatin A Nexturastat A	54, 55, 56, 57
	HDAC1	*p21* promoter	senescence bypass	Corin 69	58
	KDM1A, KDM4C	demethylation of H3K9	favors melanomagenesis by senescence bypass	GSK2879552, IOX1 68 Corin 69	68
	HDAC3	High nuclear staining	associated with improved survival of patients with stage IV metastatic melanoma	Entinostat 64	59
	HDAC8	High cytoplasmic staining	-associated with improved survival of patients with stage IV metastatic melanoma -regulates MAPK and AP-1 signaling	PCI-34051 59	59 60
	KDM5B	demethylation of H3K4	critical for melanoma tumor growth	NA	66
	KDM6B	demethylation of H3K27	upregulates several targets of NF-κB and BMP to promote melanoma progression and metastasis	GSK-J4	71
***Chromatin remodeling*** ***complexes:***	ARID2, ARID1A ARID1B, SMARCA4	chromatin remodeling	tumor suppressor?	NA	72, 73
	SMARCA4, SMARCA2	chromatin remodeling	required for melanoma tumorigenicity	NA	74
	SMARCA4	-Recruited by MITF and SOX10 to a subset of MITF-associated regulatory elements (MAREs) at active enhancers -Regulates MITF dynamics genomic occupancy	-essential for transcription regulation in melanocyte and melanoma cell physiology -progression of oncogenic Braf-driven mouse melanoma	NA	75, 76
	BPTF	chromatin remodeling essential for the melanocyte gene expression program	-regulates proliferation, migration and morphology of murine melanoblasts *in vivo* -essential for differentiation of adult melanocyte stem cells -progression of oncogenic Braf-driven mouse melanoma	NA	76, 77
	ATRX	chromatin remodeling	decreased ATRX expression correlates with melanoma progression	NA	83
***Histone variants:***	macroH2A	replace canonical H2A associated with transcription repression	-macroH2A suppresses melanoma progression via transcriptional repression of CDK8 -macroH2A loss promotes tumor growth and metastatic potential	NA	85
	H3.3	replace canonical H3	overexpression triggers senescence via E2F target genes repression	NA	86
	H2A.Z.2	replace canonical H2A, binds and stabilizes BRD2	H2A.Z.2 correlates with poor patient survival and promotes cell cycle progression via E2F target genes transcription control	NA	87

**Table 2:** Epigenetic players and their impact on melanoma resistance to therapies

	Players	Therapies	Mechanisms of resistance	Alternative treatments proposed in the study	References
**Targeted therapies**	KDM5A demethylation of H3K4	Pan-RAF inhibitor (AZ628)	Elevated expression in drug-tolerant cells and IGF-1R signaling activation	combined with IG1-1Ri (AEW541)	110
	KDM5B demethylation of H3K4	Vemurafenib	Elevated expression in slow cycling cells with increase in oxidative phosphorylation	combined with mitochondrial oxidative-ATP-synthesis (e.g. oligomycin, Bz-423)	67
	KDM1B, KDM5A, KDM5B demethylation of H3K4 KDM6A, KDM6B demethylation of H3K27	Vemurafenib or Trametinib	Elevated expression in induced drug-tolerant cells (IDTC) and undifferentiated state transition which increases aggressiveness	combined with HDACi, IGF-1Ri, PI3/AKTi to eliminate parental cells prior transition to IDTC	111
	KDM5B demethylation of H3K4	Vemurafenib or Vemurafenib+Trametinib	Elevated expression to shift into a drug-tolerant state (e.g. decrease in *Gdf15*, *Ldlr* epxression)	combined with pan-KDM5i (e.g. KDM5-C70, CPI-48)	112
	KDM6B demethylation of H3K27 EZH2 methylation of H3K27	Vemurafenib	Involved in glutamin-induced histone methylation impacting vemurafenib response	combined with EZH2i?	70
	TADA2B, TADA1 acetylation of histones H3 and H4	Vemurafenib	Loss promotes resistance; Mechanisms unknown Impacting histone acetylation and gene expression?	combined with HDACi	114
	SIRT1	Vemurafenib	Elevated expression in vemurafenib resistant cells	combined with SIRT1i (e.g. sirtinol, EX-527)	116
	SIRT2	Vemurafenib or Selumetinib	SIRT2 knockdown increases ERK signaling	NA	117
	SIRT6	Dabrafenib or Dabrafenib+Trametinib	SIRT6 haploinsufficiency activates the IGF1-R and downstream AKT signaling	combined with IGF-1Ri (i.e. linsitinib)	119
**Immunotherapies**	EZH2 methylation of H3K27	anti-CTLA4 and IL-2	Increased EZH2 activity dependent on T cells and TNF-α promoting dedifferentiation, loss of immunogenicity and PD-1/PD-L1 axis upregulation	combined with EZH2i (i.e. GSK503)	134
	ARID2, PBRM1, BRD7	anti-PD1/CTLA4 T-cell mediating killing	e.g. regulates mTORC signaling pathway	NA	135
	LSD1	anti-PD1	Represses endogenous retroviral element and interferon response with inhibition of tumor responses to host immunity	combined with GSK-LSD1	136

**Table 3:** Epigenetic drugs in combination with immunotherapy agents in melanoma

HDACi	Combination	Phase clinical trial	Identifier
Panobinostat (LBH589)	Ipilimumab	I	NCT02032810
Entinostat (MS275-SNDX-275)	Pembrolizumab	II	NCT02697630
HBI-8000	Nivolumab	I/II	NCT02718066
4SC202	Pembrolizumab	I/II	NCT03278665
CPI-1205	Ipilimumab	I/II	NCT03525795

NIH clinical trial database: www.clinicaltrials.gov
